# Treatment discontinuation, patient-reported toxicities and quality-of-life by age following trastuzumab emtansine or paclitaxel/trastuzumab (ATEMPT)

**DOI:** 10.1038/s41523-022-00495-x

**Published:** 2022-11-30

**Authors:** Tal Sella, Yue Zheng, Nabihah Tayob, Kathryn J. Ruddy, Rachel A. Freedman, Chau Dang, Denise Yardley, Steven J. Isakoff, Vicente Valero, Michelle DeMeo, Harold J. Burstein, Eric P. Winer, Antonio C. Wolff, Ian Krop, Ann H. Partridge, Sara M. Tolaney

**Affiliations:** 1 Dana-Farber Cancer Institute, Boston, MA USA; 2 Harvard Medical School, Boston, MA USA; 3 Mayo Clinic, Rochester, MN USA; 4 Department of Medicine, Memorial Sloan Kettering Cancer Center, New York, NY USA; 5 Sarah Cannon Research Institute and Tennessee Oncology, Nashville, TN USA; 6 Massachusetts General Hospital, Boston, MA USA; 7 The University of Texas MD Anderson Cancer Center, Houston, TX USA; 8 Johns Hopkins Sidney Kimmel Cancer Center, Washington, DC, USA; 9 Present Address: Department of Oncology, Sheba Medical Center, Ramat Gan, Israel; 10 Present Address: Yale Cancer Center, New Haven, CT USA

**Keywords:** Cancer, Prognosis

## Abstract

In the ATEMPT trial, adjuvant trastuzumab emtansine (T-DM1) compared to paclitaxel plus trastuzumab (TH) for stage I HER2-positive breast cancer improved patient-reported outcomes (PROs), while maintaining excellent disease outcomes. We report treatment discontinuation and use multivariable models to compare, patient-reported toxicity and quality-of-life (QOL) by age (≤50, >50) and treatment arm at 18 months post-enrollment among 366 eligible participants randomized in a 3:1 ratio to T-DM1 or TH. T-DM1 discontinuation was higher among women >50 vs. ≤50 (23% vs. 9%, *p* = 0.003, Fisher’s Exact test) with 4%, 8%, and 17% of older patients discontinuing treatment by 3, 6, and 9 months, respectively. Superior QOL with T-DM1 vs. TH was observed among women ≤50 with estimated mean difference of 6.48 (95% confidence interval (CI) 0.51–12.46) and driven by better social/family well-being and breast cancer-specific sub-scores. Among women >50, T-DM1 was associated with superior physical well-being and less activity impairment, with no differences in global QOL. Older women had decreased neuropathy with T-DM1 vs. TH. De-escalated treatment regimens for HER2 positive breast cancer may have age-varying impact on treatment tolerance, toxicities and subsequent QOL, which should be considered when selecting therapy options.

**Clinical Trial Registration:** ClinicalTrials.gov, NCT01853748

## Introduction

Trastuzumab is a well-established treatment for human epidermal growth factor receptor 2 (HER2)-positive early breast cancers. Given its high efficacy, recent efforts have concentrated on de-escalation of historic multi-agent chemotherapy protocols to safer and shorter regimens preserving previous achievements in long-term survival, while improving short and long-term quality of life (QOL)^1^. Progress is most evident for stage I HER2-positive breast cancers, with the single-arm APT trial showing excellent long-term outcomes with adjuvant paclitaxel plus trastuzumab (TH), omitting doxorubicin and cyclophosphamide^2,3^.

To further improve QOL outcomes in these patients, we conducted the ATEMPT trial, a multicenter, investigator-initiated randomized phase II study comparing a year of adjuvant T-DM1 (trastuzumab emtansine) to TH for toxicity and establishing the disease-free survival for one year of adjuvant T-DM1^4^. While T-DM1 was associated with excellent 3-year invasive disease-free survival (iDFS, 97.8% [95% confidence interval (CI), 96.3–99.3]), the co-primary outcome, a prospectively defined composite outcome including clinically relevant toxicities seen with either treatment, was equivalent (46% T-DM1 vs. 47% TH, *p* = 0.83). T-DM1 was associated with a high rate of treatment discontinuation due to adverse events (17%); however, adverse event profiles, assessed by patient-reported outcomes (PROs), revealed better QOL, lower risk of neuropathy and superior work productivity with T-DM1 vs. TH.

While the ATEMPT trial supports the use of T-DM1 as a potential adjuvant systemic therapy in stage I HER2 + breast cancers, it remains unclear which patients stand to benefit from this de-escalation^5^. Patient age is an acknowledged factor in breast cancer therapy decision-making, in some instances driving over-treatment of younger patients and undertreatment of older patients^6^. Age may also be associated with development of negative physical and emotional sequelae following breast cancer. Young survivors are consistently found to be at higher risk for adverse physical and psychological effects which may impair their QOL for years following diagnosis^7–10^. When comparing age groups, several studies show worse QOL and increased symptom burden in younger survivors, primarily in early years post-diagnosis^11–14^. However, QOL deterioration is also observed in older populations, particularly those with comorbidities less common among younger women^15^.

Given these considerations, the aim of this unplanned post-hoc analysis is to compare rates of treatment discontinuation, and patient-reported QOL and toxicities between younger and older women in ATEMPT at 18 months post-enrollment. This timepoint, 6 months after completion of all protocol therapy, provides important information regarding women’s experience as they transition to breast cancer survivorship care following one year of adjuvant therapy.

## Results

### Patient characteristics

**Fig. 1:**
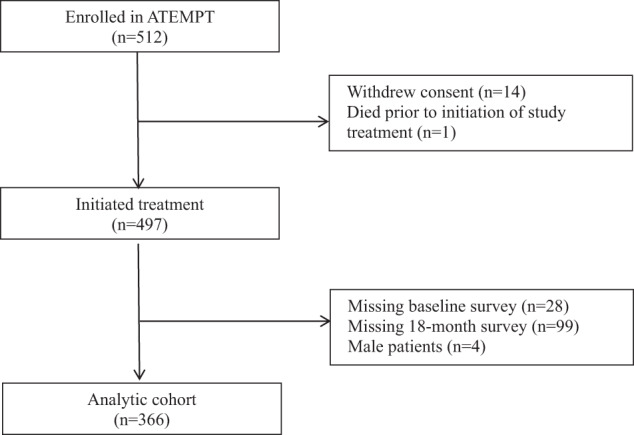
Flow diagram of participants. Of 512 participants randomized to receive treatment with either adjuvant T-DM1 or TH, 366 were included in the current analytic cohort.

Of 512 participants recruited, 497 initiated study treatment. Following exclusion of participants without a baseline (*n* = 28) or 18-month assessment (*n* = 99), and male participants (*n* = 4), 366 patients were included in this analysis (Fig. 1). Among included patients, 34% (*n* = 124) were ≤50 years and 66% (*n* = 242) were >50 with an equal distribution observed for excluded patients (34 and 66%, respectively). Additional characteristics were similar for included and excluded patients (see Supplementary Table 1 in the Supplementary File).

In the analytic cohort (*N* = 366), overall median age was 56.69 (range 23.2–85.9), 45.37 (23.2–50.9) in women ≤50 and 61.13 (51.2–85.9) in women >50 (Table 1). Treatment distribution was similar and consistent with the 3:1 allocation, with 75% of women ≤50 and 79% women >50 randomized to T-DM1 (*p* = 0.428, Fisher’s Exact test). Younger women were more commonly premenopausal at enrollment (86% vs. 11%, *p* < 0.001, Fisher’s Exact test). Tumor characteristics were similar; however, younger women more frequently underwent mastectomy (53% vs. 28%, *p* < 0.001, Fisher’s Exact test) and accordingly received less adjuvant radiotherapy (50% vs. 73%, *p* < 0.001, Fisher’s Exact test). Among patients with hormone receptor-positive (≥1% estrogen receptor expression) disease, endocrine therapy utilization at 18 months was similar between age groups (≤50: 87% vs. >50: 83%, *p* = 0.385, Fisher’s Exact test) and between arms (T-DM1: 85% vs. TH: 83%, *p* = 0.840, Fisher’s Exact test).

**Table 1:** Baseline characteristics by age group.

Characteristic	Total Population	≤50 years	>50 years	*p*-value
*n*	366	124	242	
Age (years)				
Mean (SD)	56.11 (10.5)	44.60 (5.2)	62.01 (7.1)	NA
Median (range)	56.69 (23.2, 85.9)	45.37 (23.2, 50.9)	61.13 (51.2, 85.9)	–
Race (*n*, %)				
Asian	18 (5%)	8 (6%)	10 (4%)	–
Black	22 (6%)	8 (6%)	14 (6%)	–
White	307 (84%)	100 (81%)	207 (86%)	0.584
More than one/other/unknown	19 (5%)	8 (6%)	11 (5%)	–
Ethnicity (n, %)				
Hispanic	7 (2%)	2 (2%)	5 (2%)	0.730
Non-Hispanic	332 (91%)	111 (90%)	221 (91%)	–
Unknown	27 (7%)	11 (9%)	16 (7%)	–
Baseline menopausal status (n, %)				
Premenopausal	133 (36%)	107 (86%)	26 (11%)	<0.001
Postmenopausal	233 (64%)	17 (14%)	216 (89%)	–
Tumor size (*n*, %)				
<0.5 cm	42 (11%)	16 (13%)	26 (11%)	0.839
0.5–1.0 cm	119 (33%)	37 (30%)	82 (34%)	–
1.01–1.5 cm	110 (30%)	38 (31%)	72 (30%)	–
1.51–2.0 cm	95 (26%)	33 (27%)	62 (26%)	–
Treatment arm (*n*, %)				
TH	82 (22%)	31 (25%)	51 (21%)	0.428
T-DM1	284 (78%)	93 (75%)	191 (79%)	–
ER/PR expression (*n*, %)				
Negative (<1%)	90 (25%)	29 (23%)	61 (25%)	0.143
Low Positive (1–9%)	24 (7%)	4 (3%)	20 (8%)	–
Positive (≥10%)	252 (69%)	91 (73%)	161 (67%)	–
Surgery type (*n*, %)				
Lumpectomy	231 (63%)	58 (47%)	173 (71%)	<0.001
Mastectomy	134 (37%)	66 (53%)	68 (28%)	–
Unilateral	74 (20%)	31 (25%)	43 (18%)	–
Bilateral	57 (16%)	33 (27%)	24 (10%)	–
Unknown type	3 (1%)	2 (2%)	1 (0.4%)	–
Missing	1 (0.3%)	0	1 (0.4%)	–
Radiotherapy (*n*, %)				
Yes	238 (65%)	62 (50%)	176 (73%)	<0.001
No	126 (34%)	62 (50%)	64 (26%)	–
Missing	2 (1%)	0 (0%)	2 (1%)	–
Endocrine therapy at 18 months (*n*, %)				
Yes	235 (64%)	85 (69%)	150 (62%)	0.250
No	131 (36%)	39 (31%)	92 (38%)	–

*P*-values derived from Student’s Exact test.

*SD* standard deviation, *NA* not applicable, *ER* estrogen receptor, *PR* progesterone receptor, *T-DM1* trastuzumab emtansine, *TH* paclitaxel plus trastuzumab.

### Treatment discontinuation and dose reduction

**Fig. 2:**
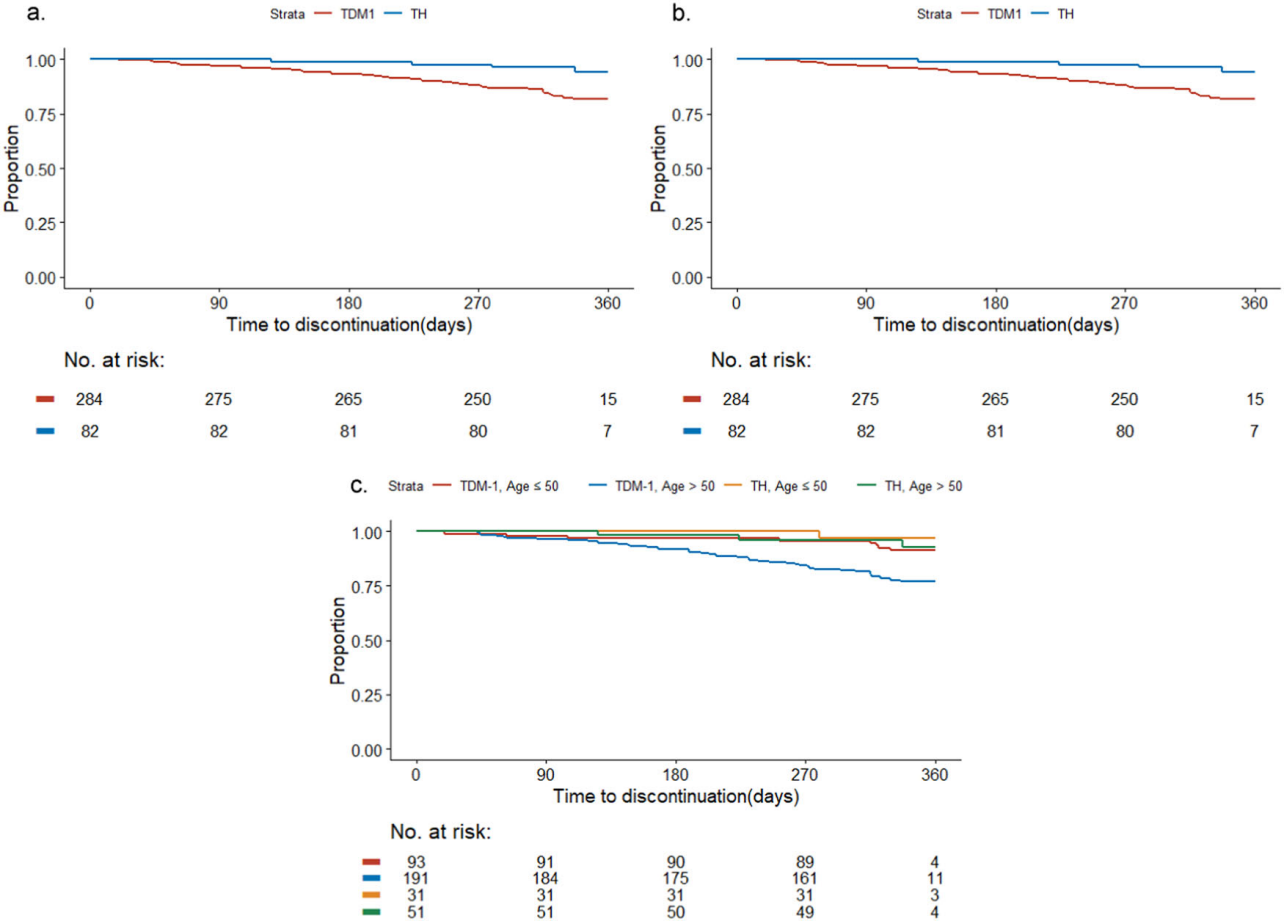
Time to discontinuation by age groups and arms. **a** Shows the time to discontinuation by age group (*p* = 0.002); **b** shows the time to early discontinuation by arm (*p* = 0.007); and **c** shows the time to early discontinuation by age group and arm (*p* < 0.001).

Discontinuations of all protocol therapy were 6 and 18% for TH and T-DM1, respectively. T-DM1 discontinuation was significantly higher among women >50 vs. ≤50 (23% vs. 9%, *p* = 0.003, Fisher’s Exact test) with 4%, 8%, and 17% of older patients discontinuing treatment by 3, 6, and 9 months, respectively (Table 2). Similarly, different rates were observed in extreme age groups: ≤40 years (5%, 1/19), ≥70 years (23%, 6/26). Time to discontinuation was significantly shorter for older women vs. younger women (*p* = 0.002, Log-rank test, Fig. 2a) and for T-DM1 vs. TH (*p* = 0.007, Log-rank test, Fig. 2b). Older women receiving T-DM1 were at particular risk for discontinuation, while discontinuation for younger women receiving T-DM1 and both age groups receiving TH, was comparable (*p* < 0.001, Log-rank test, Fig. 2c). Following T-DM1 discontinuation, 25% (2/8) of younger and 45% (20/44) of older women switched to trastuzumab to complete a year of treatment.

**Table 2:** Reasons for trastuzumab emtansine (T-DM1) discontinuation by age group.

Reason for discontinuation	≤50 years	>50 years
*n*	93	191
Discontinuation for any reason	8 (9)	44 (23)
Patient withdrew consent (*n*, %)	–	4 (2)
Unacceptable toxicity	7 (8)	35 (18)
Physician decision	4	18
Protocol-mandated	3	17
Other	1 (1)	5 (3)
Cumulative discontinuation through 1 year (*n*, %)		
0–3 months	2 (2)	7 (4)
3–6 months	3 (3)	16 (8)
6–9 months	4 (4)	32 (17)
9–12 months	8 (9)	44 (23)
Reasons for discontinuation due to toxicity (*n*, %)^a^		
Liver enzyme elevation/bilirubin elevation	2 (29)	10 (29)
Neuropathy	1 (14)	6 (17)
Platelet count decreased	1 (14)	6 (17)
Bleeding	–	2 (6)
Cardiotoxicity	–	2 (6)
Cough/dyspnea	2 (29)	–
Nausea/vomiting	–	3 (9)
Anemia	1 (14)	–
Eye disorders - Other, specify	–	1 (3)
Fatigue	–	1 (3)
Headache	–	1 (3)
Myalgia	–	1 (3)
Pneumonitis	–	1 (3)
Telangiectasia	–	1 (3)

^a^Percentage from discontinuations due to toxicity.

Toxicity was the primary reason for T-DM1 discontinuation and was higher among older women (18% vs. 8%). In both age groups approximately half of discontinuations were protocol-mandated and half based on the treating physician’s decision (Table 2). Among older women, the most common toxicities for T-DM1 discontinuation were elevated liver enzymes or bilirubin (29%), neuropathy (17%), and thrombocytopenia (17%). Discontinuations due to cardiotoxicity were infrequent (*n* = 2) and limited to the older subgroup.

T-DM1 dose reductions occurred in 18% of women included in this analysis and were more common in older compared to younger women, (20%, 38/128 vs. 14%, 13/93, respectively, *p* = 0.011, Fisher’s Exact test).

### Patient-reported outcomes

PRO scores at baseline and 18-months are summarized by arm and age group (Table 3). In multivariable analysis, better 18-month Functional Assessment of Cancer Therapy-Breast Cancer (FACT-B)^16^ total score was associated with better baseline FACT-B total score (estimated mean difference 0.73, *p* < 0.001, linear regression), but not with age or treatment arm (Table 4). Independent associations between 18-month FACT-B and age or arm were not found; however, an interaction between the two was observed (*p* = 0.037, linear regression): among women ≤50, treatment with T-DM1 (vs. TH) was associated with better 18-month FACT-B total score with an estimated mean difference of 6.48 (95% CI 0.51–12.46), approaching the minimally important difference (MID) threshold of 7–8 points^17^. Additionally, within the T-DM1 group, younger age was associated with better adjusted 18-month FACT-B total scores than older age (estimated mean difference in score between age ≤50 and age >50, 4.12; 95% CI 0.32–7.92). We performed a sensitivity analysis replacing the dichotomized age variable with baseline menopausal status (premenopausal or postmenopausal). In contrast to the primary model which showed a significant interaction between age group and treatment arm, in the sensitivity model, the interaction between baseline menopausal status and arm was not significant (*p* = 0.993, linear regression).

**Table 3:** Unadjusted patient-reported outcomes by study arm and age group at baseline and 18-months post-enrollment.

Patient-reported outcome measure	Baseline				18 months			
	TH		T-DM1		TH		TDM1	
	≤50	>50	≤50	>50	≤50	>50	≤50	>50
FACT-B (total) - mean (SD)								
*n*	30	51	93	188	31	48	92	188
FACT-B total score	115.06 (20.58)	116.53 (20.07)	122.25 (14.40)	128.35 (13.63)	114.59 (24.57)	119.69 (23.00)	126.20 (13.46)	126.38 (16.72)
Physical well-being	25.57 (3.82)	26.02 (3.26)	26.14 (2.62)	26.69 (3.07)	23.84 (5.24)	23.58 (4.83)	25.77 (2.74)	25.21 (3.93)
Social/family well-being	23.89 (6.13)	24.64 (4.11)	24.98 (4.85)	25.88 (3.94)	21.90 (7.37)	24.34 (6.07)	24.81 (3.80)	24.77 (5.47)
Emotional well-being	16.83 (4.63)	17.42 (4.46)	19.00 (3.46)	19.69 (2.95)	18.41 (4.62)	19.46 (4.80)	20.42 (3.30)	20.76 (2.86)
Functional well-being	20.03 (5.41)	20.35 (6.30)	20.03 (5.41)	23.68 (4.48)	21.34 (7.13)	22.37 (6.01)	23.88 (4.49)	23.43 (5.35)
Breast cancer subscale	28.73 (6.68)	28.26 (7.00)	28.73 (6.68)	32.33 (4.57)	28.18 (7.70)	30.06 (6.13)	31.34 (4.81)	32.08 (5.15)
RSCL - mean (SD)								
*n*	30	51	92	188	31	47	91	186
Physical symptom distress	30.61 (7.31)	30.96 (6.83)	28.95 (4.95)	27.80 (5.09)	32.10 (8.97)	32.50 (7.97)	30.36 (5.97)	31.29 (7.60)
Psychological distress	14.73 (5.13)	14.80 (4.99)	12.65 (4.23)	10.93 (3.23)	12.23 (4.33)	11.13 (4.70)	10.14 (3.83)	9.73 (3.22)
Activity level	30.92 (2.30)	30.82 (2.72)	30.36 (4.01)	31.24 (1.94)	31.41 (1.46)	30.34 (3.69)	31.61 (1.82)	30.75 (3.61)
Overall valuation of life	1.81 (1.04)	2.14 (1.53)	1.82 (0.95)	1.48 (0.72)	2.00 (1.41)	2.10 (1.30)	1.64 (0.89)	1.71 (0.94)
WPAI:SHP - mean (SD)								
*n*	30	50	91	188	31	44	91	184
Percentage employed - (*n*, %)	20 (67%)	24 (48%)	75 (83%)	99 (53%)	24 (77%)	21 (48%)	76 (84%)	91 (48%)
% work time missed due to breast cancer	29.05 (38.63)	17.02 (25.19)	34.56 (41.48)	14.92 (28.28)	6.11 (21.11)	2.22 (5.09)	3.53 (14.45)	2.53 (12.58)
% impairment at work due to breast cancer	14.67 (19.95)	24.21 (29.12)	19.62 (26.19)	5.60 (10.79)	7.39 (18.15)	8.24 (15.51)	3.53 (9.58)	2.44 (7.35)
% overall work impairment due to breast cancer	27.01 (27.13)	32.78 (31.53)	29.26 (32.91)	14.61 (22.17)	9.30 (19.09)	9.96 (17.25)	5.47 (12.91)	3.72 (10.42)
% activity impairment due to breast cancer	23.45 (27.68)	18.98 (24.94)	21.41 (27.40)	10.82 (18.92)	12.67 (26.38)	16.19 (28.71)	6.44 (16.44)	7.43 (16.53)
APA - (*n*, %)								
*n*	29	49	93	183	31	44	88	177
Any hair loss	1 (3%)	5 (10%)	3 (3%)	15 (8%)	3 (10%)	9 (20%)	12 (14%)	23 (13%)
PNQ - (*n*, %)								
*n*	30	51	92	182	31	48	92	189
Any moderate, moderate-severe or severe neuropathy	3 (10%)	1 (2%)	7 (8%)	3 (2%)	8 (26%)	19 (40%)	9 (10%)	39 (21%)

*SD* standard deviation, *T-DM1* trastuzumab emtansine, *TH* paclitaxel plus trastuzumab, *FACT-B* Functional Assessment of Cancer Therapy-Breast Cancer, *RSCL* Rotterdam Symptom Checklist, *WPAI:SHP* Work Productivity and Activity Impairment Questionnaire: Specific Health Problem, *APA* Alopecia Patient Assessment, *PNQ* Patient Neurotoxicity Questionnaire.

**Table 4:** Multivariable linear regression model for mean difference in 18-month post-enrollment FACT-B total score.

Covariate	Estimate	95% CI	SE	*p*-value
Intercept	36.37	19.38, 53.37	8.64	<0.001
Age ≤50 (vs. >50)	−3.87	−10.52, 2.77	3.38	0.252
Arm – T-DM1 vs. TH	−1.51	−6.31, 3.29	2.44	0.536
Age (≤50) * Arm (T-DM1)	7.99	0.47, 15.51	3.82	0.037
≤50 vs. >50, arm = T-DM1	4.12	0.32, 7.92	1.93	0.034
≤50 vs. >50, arm = TH	−3.87	−10.52, 2.77	3.38	0.252
T-DM1 vs. TH, age ≤50	6.48	0.51, 12.46	3.04	0.034
T-DM1 vs. TH, age >50	−1.51	−6.31, 3.29	2.44	0.536
Baseline FACT-B total score	0.73	0.63, 0.83	0.05	<0.001
Early discontinuation of TH/T-DM1 (vs. no)	−0.82	−5.17, 3.53	2.21	0.711
Race (vs. white)				
Asian	1.60	−5.46, 8.66	3.59	0.656
Black	−5.89	−12.59, 0.82	3.41	0.085
Other	0.19	−6.55, 6.94	3.43	0.955
Mastectomy (vs. lumpectomy)	−0.29	−11.74, 11.16	5.82	0.960
Receipt of radiotherapy (vs. no)	−2.29	−13.86, 9.28	5.88	0.697
Taking endocrine therapy 18 months (vs. no)	0.37	−2.79, 3.53	1.61	0.819

Variables tested include age group, treatment arm, and their interaction term (arm*age group), race, early discontinuation, surgery type, receipt of radiotherapy, and endocrine therapy use at 18 months and the respective PRO score at baseline. A positive estimate indicates a higher (superior) FACT-B total score in the first comparator group vs. the second. *P*-value is derived from the linear regression model.

*FACT-B* Functional Assessment of Cancer Therapy-Breast Cancer, *CI* confidence interval, *SE* standard error, *T-DM1* trastuzumab emtansine, *TH* paclitaxel plus trastuzumab.

Table 5 and Supplementary Fig. 1 (in the Supplementary File) list adjusted mean differences in 18-month PROs for age groups and treatment arms (interaction). When controlling for baseline values of the outcome measure and other covariates, higher 18-month FACT-B total scores among younger women treated with T-DM1 vs. TH were driven by differences in social/family well-being (SWB) (estimated mean difference in score between T-DM1 and TH, 2.61; 95% CI 0.64–4.58) and breast cancer subscale (BCS) (estimated mean difference in score between T-DM1 and TH, 1.92; 95% CI 0.05–3.79) sub-scores. T-DM1 was significantly associated with better physical well-being (PWB) scores vs. TH in women >50 (estimated mean difference in score between T-DM1 and TH, 1.43; 95% CI 0.26–2.60). A similar non-significant point estimate was found for women ≤50 (estimated mean difference in score between T-DM1 and TH, 1.35; 95% CI −0.15–2.86). All significant inter-group differences met or approached MID threshold (2–3 points for BCS, 1–3 points for PWB)^17,18^.

**Table 5:** Adjusted differences between age groups and treatment arms (interaction) in 18-month post-enrollment patient-reported outcomes.

Patient-reported outcome measure	Comparing age groups by arms ≤50 vs. >50		Comparing arms by age groups T-DM1 vs. TH		*P*_interaction_
	TH	T-DM1	≤50	>50	
	Mean difference, 95% confidence interval, *p*-value				
FACT-B (positive score indicates superior outcome for first comparator group)					
FACT-B total score	−3.87 (−10.52, 2.77)	4.12 (0.32, 7.92)	6.48 (0.51, 12.46)	−1.51 (−6.31, 3.29)	0.037
Physical well-being	0.87 (−0.82, 2.55)	0.79 (−0.15, 1.73)	1.35 (−0.15, 2.86)	1.43 (0.26, 2.60)	0.938
Social/family well-being	−2.03 (−4.24, 0.18)	0.69 (−0.55, 1.93)	2.61 (0.64, 4.58)	−0.11 (−1.64, 1.43)	0.032
Emotional well-being	−0.18 (−1.60, 1.24)	0.17 (−0.62, 0.96)	0.54 (−0.74, 1.82)	0.20 (−0.82, 1.21)	0.668
Functional well-being	−0.90 (−3.20, 1.40)	1.41 (0.12, 2.70)	1.82 (−0.24, 3.88)	−0.49 (−2.13, 1.16)	0.081
Breast cancer subscale	−2.11 (−4.21, −0.01)	0.23 (−0.95, 1.41)	1.92 (0.05, 3.79)	−0.41 (−1.95, 1.12)	0.054
RSCL (positive score (except otherwise noted) indicates inferior outcome for first comparator group)					
Physical symptom distress	−1.07 (−3.85, 1.72)	−1.47 (−3.05, 0.11)	0.14 (−2.36, 2.64)	0.54 (−1.44, 2.52)	0.802
Psychological distress	0.39 (−1.10, 1.88)	−0.45 (−1.30, 0.41)	−0.65 (−2.00, 0.69)	0.19 (−0.91, 1.29)	0.330
Activity level^a^	1.06 (−0.42, 2.53)	1.10 (0.28, 1.93)	0.44 (−0.89, 1.77)	0.40 (−0.62, 1.41)	0.959
Overall valuation of life	0.09 (−0.40, 0.58)	−0.22 (−0.49, 0.06)	−0.32 (−0.77, 0.12)	−0.02 (−0.35, 0.32)	0.279
WPAI:SHP (positive score indicates inferior outcome for first comparator group)					
% work time missed due to breast cancer	−3.80 (−14.47, 6.86)	−1.60 (−6.58, 3.37)	2.39 (−5.17, 9.94)	0.18 (−8.77, 9.14)	0.708
% impairment at work due to breast cancer	0.13 (−8.70, 8.97)	−2.19 (−6.48, 2.10)	−4.95 (−11.75, 1.84)	−2.63 (−9.83, 4.57)	0.642
% overall work impairment due to breast cancer	−1.73 (−12.07, 8.61)	−2.65 (−7.58, 2.29)	−3.59 (−11.39, 4.20)	−2.68 (−11.21, 5.85)	0.875
% activity Impairment due to breast cancer	−4.63 (−13.33, 4.07)	−3.77 (−8.58, 1.04)	−5.68 (−13.32, 1.96)	−6.53 (−12.79, −0.28)	0.864
Odds ratio, 95% confidence interval, *p*-value					
APA – any hair loss	0.39 (0.07, 2.13)	1.23 (0.54, 2.84)	1.95 (0.39, 9.68)	0.62, (0.24, 1.58)	0.225
PNQ - any moderate, moderate-severe or severe neuropathy	0.46 (0.15, 1.38)	0.44 (0.19, 1.02)	0.32 (0.10, 1.03)	0.33 (0.16, 0.68)	0.951

Table shows interactions terms for arm*age-group taken from linear (RSCL, WPAI, FACT-B) and logistic (APA, PNQ) multivariable regression models. Values are mean differences/odds ratios measured between age-groups (within each arm) or between arms (within each age group) and respective interaction p-values. Variables tested include age-group, arm, arm*age-group, race, early discontinuation, surgery type, receipt of radiotherapy, receipt of endocrine therapy at 18 months and the respective PRO score at baseline.

*T-DM1* trastuzumab emtansine, *TH* paclitaxel plus trastuzumab, *FACT-B* Functional Assessment of Cancer Therapy-Breast Cancer, *RSCL* Rotterdam Symptom Checklist, *WPAI:SHP* Work Productivity and Activity Impairment Questionnaire: Specific Health Problem, *APA* Alopecia Patient Assessment, *PNQ* Patient Neurotoxicity Questionnaire.

^a^higher score indicates higher (better) activity level and positive score indicates superior outcome for first comparator group.

Adjusted 18-month Rotterdam Symptom Checklist (RSCL)^19,20^ scores were comparable between age groups and arms; only activity level was significantly worse in younger vs. older women treated with T-DM1 (estimated mean difference in score between age ≤50 and age >50, 1.10; 0.28–1.93), although a similar point estimate was seen after TH (1.06, −0.42–2.53).

Using the 18-month Work Productivity and Activity Impairment Questionnaire: Specific Health Problem (WPAI:SHP)^21^, among women >50 years, T-DM1 vs. TH was associated with less activity impairment due to breast cancer (estimated mean difference in score between T-DM1 and TH, −6.53; 95% CI −12.79 to −0.28). No additional differences in mean WPAI:SHP scores were observed.

Adjusted odds of alopecia at 18 months, as reported on the Alopecia Patient Assessment (APA)^22^, did not significantly differ by age or arm. Using the Patient Neurotoxicity Questionnaire (PNQ)^23^, adjusted odds of 18-month residual moderate, moderate-severe or severe neuropathy were significantly lower with T-DM1 vs. TH among women >50 (odds ratio [OR] 0.33, 95% CI 0.16–0.68) with a trend for reduction among women ≤50 (OR 0.32, 95% CI 0.10–1.03).

## Discussion

In light of favorable disease-related outcomes seen in contemporary trials for HER2-positive early breast cancer, ongoing efforts increasingly emphasize treatment de-escalation as a means of optimizing health-related quality of life (HRQOL) while sustaining treatment efficacy. In ATEMPT, adjuvant T-DM1 was associated with superior overall HRQOL, lower risk of neuropathy and superior work productivity, while maintaining excellent 3-year iDFS in patients with stage I HER2-positive breast cancer^4^. Analogous findings were reported in the KAITLIN trial, comparing similar regimens (plus pertuzumab) for stage II-III disease though following anthracycline-based chemotherapy^24^. Our current analysis shows that younger women, while opting for more aggressive surgery, and more often completing protocol therapy than older women, report larger HRQOL gains at 18 months with T-DM1 vs. TH, with differences within or approaching the range of clinical relevance^17^.

Multiple studies have identified a distinct and often more severe impact of breast cancer on younger survivors’ HRQOL and emotional well-being^7–9,13^. In a systematic review comparing younger (≤50) to older women (>50), HRQOL was more severely compromised in younger women, with greater deterioration noted for mental health as opposed to physical functioning domains^9^. In a recent longitudinal report, a steeper drop in HRQOL was observed among younger (≤50) vs. older (>50) survivors during the first three years post-diagnosis, and although HRQOL improved thereafter, at 10 years it remained below the general population level^14^. Our findings, showing better 18-month HRQOL particularly in young women treated with T-DM1 vs. TH, suggest that a modern, de-escalated chemotherapy approach may temper these effects on young women’s HRQOL. Additionally, complementary to prior studies, this improvement was driven by better SWB and BCS sub-scores, (including items focused on body image and sexuality) rather than PWB.

It is uncertain why T-DM1 led to superior 18-month HRQOL in young women. We previously showed that during the first 12 weeks of treatment, T-DM1 vs. TH was associated with less missed work time and work/activity impairment, and lower rates of alopecia and neuropathy^4^. By 18 months, these differences attenuated, although a lower risk of neuropathy following T-DM1 persists, regardless of age. Specific to younger women, treatment-related menopause is a toxicity with more long-term effects, which may in part explain our findings. In a preplanned sub-study, among premenopausal women enrolled to ATEMPT, 18-month chemotherapy-related amenorrhea was significantly lower with T-DM1 vs. TH (24% vs. 50%)^25^. Treatment-related menopause is associated with physiologic symptoms such as night sweats, hot flashes, vaginal dryness, and weight gain, which can adversely affect patients’ psychosocial QOL^26,27^. In younger premenopausal women, preserved ovarian function also contributes to fertility preservation, an important issue for many young patients^28^. In a sensitivity model however, after replacing age with baseline menopausal status, we did not replicate the significant interaction observed between age group and treatment arm.

Over half of women ≤50 treated for stage I breast cancers in ATEMPT underwent mastectomy, nearly twice the rate observed for women >50. Additionally, half of mastectomies in younger women were bilateral. These observations conform with national trends showing increasing rates of mastectomy, and particularly bilateral mastectomy, with steeper increases in younger patients and those with node-negative tumors ≤2 cm^29^. Compared to breast-conserving surgery, mastectomy with implant reconstruction is associated with inferior breast satisfaction, psychosocial well-being scores, and sexual well-being scores, even when restricting to stage I cancers^30^. Given the extremely low rates of locoregional recurrence associated with HER2-positive disease following adequate anti-HER2 therapy, less aggressive surgery may serve as an additional means to retain QOL^31^.

Among older women, we did not observe a significant difference in 18-month global HRQOL between arms, although T-DM1 was associated with better physical well-being, less activity impairment and lower odds of neuropathy. This may be partially related to increased toxicity and higher rates of T-DM1 discontinuation in older women, although we corrected for these in multivariable analyses. Additionally, our study was underpowered to examine differential treatment effects on HRQOL in women at extremes of age (≥65–70) and at higher risk of developing chemotherapy toxicity^6,32^. The ATEMPT 2.0 trial (NCT04893109) is evaluating whether six cycles of T-DM1 followed by trastuzumab can decrease toxicity while maintaining efficacy and will compare toxicities of this regimen to TH in patients with stage I HER2-positive breast cancer.

The current study’s strengths include its prospective nature and high-quality data captured within the setting of a multicenter clinical trial. We applied an age stratification (≤50, >50) commonly used in the study of breast cancer, facilitating comparisons to prior studies, but limiting our ability to comment on women at age extremes, primarily the elderly. However, the care of elderly patients can be complicated by geriatric factors and comorbidities (not captured within our data), and thus a clinical trial population may not be representative^6^. Safety and efficacy of adjuvant T-DM1 in older patients (≥60 years) with stage I-III HER2-positive breast cancer is being evaluated in the ATOP trial (NCT03587740). Generalizability of our findings may also be limited by the small number of minority participants. Racial/ethnic variations in HRQOL after breast cancer have been described, although they may be less evident in younger women due to their overall worse HRQOL^33^. Lastly, although baseline characteristics, including distribution of age groups, were similar for patients with missing surveys (*n* = 127) and the study population, we cannot exclude divergent PROs.

Our findings suggest that younger breast cancer patients, a population at times overtreated and at particular risk for QOL impairment, may benefit more than older women from use of T-DM1 rather than TH with regard to HRQOL. This is notable as it was observed with a full year of T-DM1 and compared to an already “de-escalated” regimen. Although younger patients and their providers may hesitate to accept de-escalated regimens, recent data suggest against an association between age and prognosis in adequately treated HER2-positive early breast cancer^1,34–37^. The potential for greater improvement in QOL further supports the prudent application of de-escalation strategies in the treatment of young and older breast cancer patients alike. PRO analyses, upcoming reports of longer-term outcomes from ATEMPT, and future data regarding the efficacy of a shorter course of T-DM1 from ATEMPT 2.0, will continue to shape recommendations for T-DM1 in the adjuvant setting.

## Methods

### Study population and procedures

ATEMPT (TBCRC033) was a randomized phase II trial that enrolled 512 participants within 90 days of their most recent surgery for stage I HER2-positive breast cancer at 24 institutions throughout the United States between 17 May 2013 and 13 December 2016^4^. Patients were stratified by age (<55/≥55 years), planned radiotherapy (yes/no), and planned endocrine therapy (yes/no) and randomized in a 3:1 ratio to receive T-DM1 or TH, respectively. T-DM1 (3.6 mg/kg) was administered intravenously on day 1 of each 21-day cycle and continued for 17 cycles or 1 year. TH entailed intravenous weekly administration of paclitaxel (80 mg/m2) with concurrent weekly trastuzumab (4 mg/kg loading dose followed by 2 mg/kg(for 12 weeks, with trastuzumab (6 mg/kg) subsequently continued intravenously every 21 days for 13 cycles. Adjuvant radiotherapy and hormonal therapy could be initiated after 12 weeks of T-DM1 or completing paclitaxel. Female participants completing both baseline and 18-month survey assessments were included. The study (NCT01853748) was conducted in accordance with the International Conference on Harmonization Good Clinical Practice Standards and the Declaration of Helsinki. Institutional review board (IRB) approval was obtained at Dana-Farber/Harvard Cancer Center and participating sites (see the list of participating IRBs in the Supplementary Note, included in the Supplementary File). Written informed consent was obtained from each patient. The full trial protocol is available as a supplement.

### Measures

Following randomized treatment allocation, English-speaking participants were surveyed at baseline (day 1 of treatment), 3 and 12 weeks, and 6, 12, and 18 months (24 months for QOL and symptom distress). To focus on posttreatment outcomes, the current analysis includes data collected at baseline and 18 months, the last timepoint at which all PRO instruments were administered. PRO surveys included FACT-B^16^, RSCL^20^, APA^22^, PNQ^23^, and WPAI:SHP^21^.

The FACT-B (Version 4) is designed to measure multidimensional health-related QOL (HRQOL) in breast cancer patients providing a total score (37 items, range 0–148) and 5 subscale scores: (PWB, 7 items, range 0–28), SWB, 7 items, range 0–28), emotional well-being (EWB, 6 items, range 0–24), functional well-being (FWB, 7 items, range 0–28), and (BCS, 10 items, range 0–40)^16^. Higher scores indicate better HRQOL. Differences of 7–8 points on the FACT-B score and 1–3 points for subscale scores are considered a minimally important difference (MID)^17,18^.

The RSCL measures HRQOL and symptoms in cancer patients, non-specific to breast cancer. It is comprised of four scales: physical distress (23 items, range 23–92), psychological distress (7 items, range 7–28), activity level (8 items, range 8–32), and a single item measuring overall HRQOL (range 1–7)^19^. Higher scores correlate with poor HRQOL, except for activity scale, for which higher scores correlate with better HRQOL.

The WPAI:SHP is a 6-item instrument designed to quantitatively assess the amount of absenteeism (percent work time missed), presenteeism (percent reduced on-the-job effectiveness), productivity loss and overall activity impairment attributable to a specific health problem^20^. Scores are transformed into percentages (range 0–100) with higher percentages indicating greater impairment. Missing data were handled similarly for all instruments – scores were calculated only when at least 50% of items were available and prorated for missing items. FACT-B total score was calculated only if all component subscales were valid.

Peripheral neuropathy symptoms and alopecia were assessed using specific instruments. PNQ asks participants to grade each, sensory and motor neuropathy symptoms on a 5-point Likert scale. Results were categorized as no/mild neuropathy or moderate/moderate-severe/severe neuropathy. For alopecia, we used a single item from the APA, “Have you had any hair loss during the past week?” for which participants responded “yes” or “no”.

Socio-demographic and disease characteristics were collected at enrollment. Age was dichotomized as ≤50 and >50 years, a cut-off used in other studies and approximating menopausal status^9,11,14^. Race and ethnicity were extracted from the medical record. Race was categorized as American Indian or Alaskan Native, Asian, Black or African American, Native Hawaiian or Other Pacific Islander, White, more than one, or other. Ethnicity was categorized as Hispanic or Latino, Non-Hispanic or unknown. Women reporting at least one menstrual period within 12 months prior to registration were considered premenopausal and otherwise postmenopausal. Primary breast surgery (lumpectomy/mastectomy) was defined at enrollment. Receipt of radiotherapy and endocrine therapy use were collected through follow-up.

### Statistical analysis

Patient characteristics were compared between age groups using Fisher’s exact test. Discontinuation rates, as defined in the main study protocol, were compared between arms and age groups using Kaplan–Meier curves and a log-rank test was used to compare discontinuation rates at 12 months, the duration of protocol treatment. FACT-B, RSCL and WPAI:SHP scores were expressed as means ± standard deviations. PNQ and APA results were categorized as described and reported as percentages. Linear (RSCL, WPAI:SHP, FACT-B) and logistic (APA, PNQ) multivariable regression models were used to compare PROs within age groups and arms at 18 months post-enrollment. Multivariate regression models included age group, treatment arm, and their interaction term (arm*age group) and were adjusted for covariates: race, early discontinuation, surgery type, receipt of radiotherapy and endocrine therapy use at 18 months and the respective PRO score at baseline. Regression models were implemented on non-missing data for variables entered. Models were not adjusted for multiple comparisons. All p-values were 2-sided and considered statistically significant if < 0.05. Analyses were conducted using SAS Software, Version 9.4 (SAS Institute, Cary, NC).

## Supplementary information

Supplementary Material

## Data Availability

Research data are stored in an institutional repository and will be shared upon request to the corresponding author.
