# Supplementary material for: Treatment discontinuation, patient-reported toxicities and quality-of-life by age following trastuzumab emtansine or paclitaxel/trastuzumab (ATEMPT)
Source: NPJ Breast Cancer. 2022 Nov 30;8:127. doi: 10.1038/s41523-022-00495-x (PMC9712612; doi:10.1038/s41523-022-00495-x)
Supplement: Supplementary file 1 — Supplementary Material [file 41523_2022_495_MOESM1_ESM.pdf]

Supplementary File

Treatment discontinuation, patient-reported toxicities and quality-of-life by age following trastuzumab emtansine or paclitaxel/trastuzumab (ATEMPT)

Table of Contents:

- Supplementary Table 1: Baseline characteristics for patients included and excluded from analysis
- Supplementary Figure 1: Adjusted differences between age groups and treatment arms (interaction) in 18-month post-enrollment patient-reported outcomes
- Supplementary Note: Participating Institutional Review Boards (IRBs) List

Supplementary Table 1: Baseline characteristics for patients included and excluded from analysis

Characteristics	In analysis (n=366)	Excluded (n=131)	Overall ATEMPT population (N=497)
**Age, years**			
Mean (SD)	56.11 (10.48)	56.16 (10.29)	56.13 (10.42)
Median (range)	56.69 (23.24, 85.90)	56.19 (34.05, 78.56)	56.52 (23.24, 85.90)
≤50 years	124 (34%)	45 (34%)	169 (34%)
>50 years	242 (66%)	86 (66%)	328 (66%)
**Sex (n, %)**			
Female	366 (100%)	125 (95%)	491 (99%)
Male	0 (0)	6 (5%)	6 (1%)
**Race (n, %)**			
Asian	18 (5%)	8 (6%)	26 (5%)
Black	22 (6%)	6 (5%)	28 (6%)
White	307 (84%)	113 (86%)	420 (85%)
More than one/Other/Unknown	19 (5%)	4 (3%)	23 (5%)
**Ethnicity (n, %)**			
Hispanic	7 (2%)	5 (4%)	12 (2%)
Non-Hispanic	332 (91%)	117 (89%)	449 (90%)
Unknown	27 (7%)	9 (7%)	36 (7%)
**Baseline menopausal status (n, %)**			
Premenopausal	133 (36%)	34 (26%)	167 (34%)
Postmenopausal	233 (64%)	69 (53%)	302 (61%)
Missing	0 (0%)	28 (21%)	28 (6%)
**Tumor Size (n, %)**			
≤0.1 cm	6 (2%)	5 (4%)	11 (2%)
>0.1-0.5 cm	52 (14%)	18 (14%)	70 (14%)
>0.5-1.0 cm	121 (33%)	46 (35%)	167 (34%)
>1.0-1.5 cm	117 (32%)	36 (27%)	153 (31%)
>1.5-2.0 cm	70 (19%)	26 (20%)	96 (19%)
**Treatment arm (n, %)**			
TH	82 (22%)	32 (24%)	114 (23%)
T-DM1	284 (78%)	99 (76%)	383 (77%)
**ER/PR expression (n, %)**			
Negative (<1%)	90 (25%)	34 (26%)	124 (25%)
Low Positive (1-9%)	24 (7%)	4 (3%)	28 (6%)
Positive (=>10%)	252 (69%)	93 (71%)	345 (69%)
**Surgery type (n, %)**			
Lumpectomy	231 (63%)	76 (58%)	307 (62%)
Mastectomy	134 (37%)	55 (42%)	189 (38%)
Unilateral	74 (20%)	27 (21%)	101 (20%)
Bilateral	57 (16%)	27 (21%)	84 (17%)
Unknown type*	3 (1%)	1 (0%)	4 (1%)
Missing	1 (0%)	0 (0%)	1 (0%)
**Radiotherapy (n, %)**			
Yes	238 (65%)	70 (53%)	308 (62%)
No	126 (34%)	56 (43%)	182 (37%)
Missing	2 (1%)	5 (4%)	7 (1%)
**Endocrine therapy at 18 months (n, %)**			
Yes	235 (64%)	85 (65%)	320 (64%)
No	131 (36%)	46 (35%)	177 (36%)

SD, standard deviation; ER, estrogen receptor; PR, progesterone, receptor; T-DM1, trastuzumab emtansine; TH, paclitaxel plus trastuzumab

Supplementary Figure 1:
Adjusted differences between age groups and treatment arms (interaction)
in 18-month post-enrollment patient-reported outcomes

Table shows interactions terms for arm*age-group taken from linear (RSCL, WPAI, FACT-B) and logistic (APA, PNQ) multivariable regression models. Values are mean differences/odds ratios measured between age-groups (within each arm) or between arms (within each age group) and respective interaction p-values. Variables tested include age-group, arm, arm*age-group, race, early discontinuation, surgery type, receipt of radiotherapy, receipt of endocrine therapy at 18 months and the respective PRO score at baseline. Unless otherwise noted, for FACT-B and WPAI:SHP a positive score indicates superior outcome for the first comparator group, for RSCL a positive score indicates inferior outcome for the first comparator group.

T-DM1, trastuzumab emtansine; TH, paclitaxel plus trastuzumab; FACT-B, Functional Assessment of Cancer Therapy-Breast Cancer; RSCL, Rotterdam Symptom Checklist; WPAI:SHP, Work Productivity and Activity Impairment Questionnaire: Specific Health Problem; APA, Alopecia Patient Assessment; PNQ, Patient Neurotoxicity Questionnaire

Patient-reported outcome measure	Mean difference (95% CI)
**FACT-B total score**	
TH	-3.87 (-10.5, 2.77)
TDM1	4.12 (0.32, 7.92)
**Physical well-being**	
TH	0.87 (-0.82, 2.55)
TDM1	0.79 (-0.15, 1.73)
**Social/family well-being**	
TH	-2.03 (-4.24, 0.18)
TDM1	0.69 (-0.55, 1.93)
**Emotional well-being**	
TH	-0.18 (-1.60, 1.24)
TDM1	0.17 (-0.62, 0.96)
**Functional well-being**	
TH	-0.90 (-3.20, 1.40)
TDM1	1.41 (0.12, 2.70)
**Breast cancer subscale**	
TH	-2.11 (-4.21, -0.01)
TDM1	0.23 (-0.95, 1.41)
**Physical symptom distress***	
TH	1.07 (-1.72, 3.85)
TDM1	1.47 (-0.11, 3.05)
**Psychological distress***	
TH	-0.39 (-1.88, 1.10)
TDM1	0.45 (-0.41, 1.30)
**Activity level**	
TH	1.06 (-0.42, 2.53)
TDM1	1.10 (0.28, 1.93)
**Overall valuation of life***	
TH	-0.09 (-0.58, 0.40)
TDM1	0.22 (-0.06, 0.49)
**% work time missed due to breast cancer***	
TH	3.80 (-6.86, 14.47)
TDM1	1.60 (-3.37, 6.58)
**% impairment at work due to breast cancer***	
TH	-0.13 (-8.97, 8.70)
TDM1	2.19 (-2.10, 6.48)
**% overall work impairment due to breast cancer***	
TH	1.73 (-8.61, 12.07)
TDM1	2.65 (-2.29, 7.58)
**% activity Impairment due to breast cancer***	
TH	4.63 (-4.07, 13.33)
TDM1	3.77 (-1.04, 8.58)

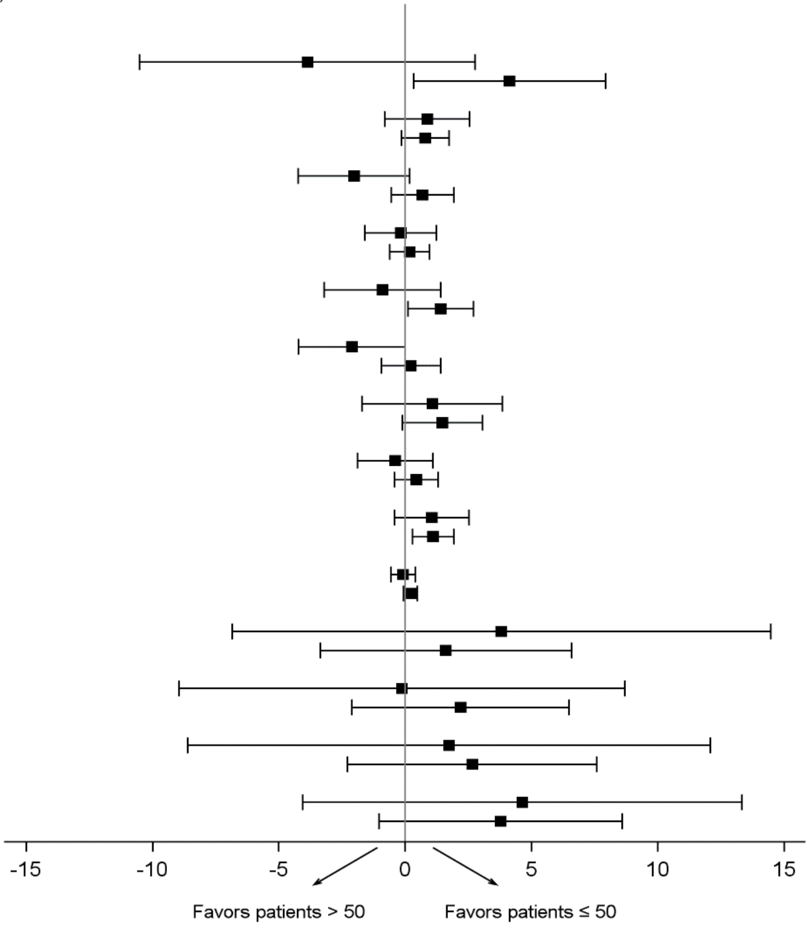

Patient-reported outcome measure	Odds Ratio (95% CI)
**APA – any hair loss***	
TH	2.56 (0.47, 13.96)
TDM1	0.81 (0.35, 1.87)
**PNQ - Any moderate, moderate-severe or severe neuropathy***	
TH	2.20 (0.72, 6.67)
TDM1	2.29 (0.98, 5.38)

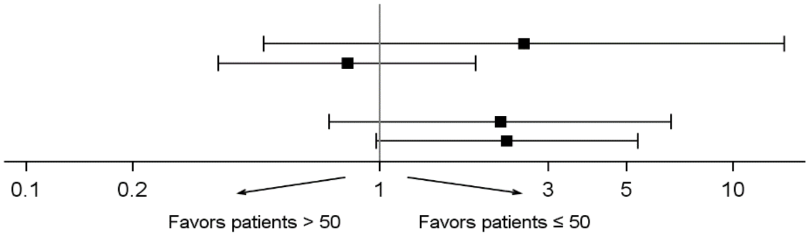

*Reference group reversed so that positive score indicates superior outcome for patients ≤ 50

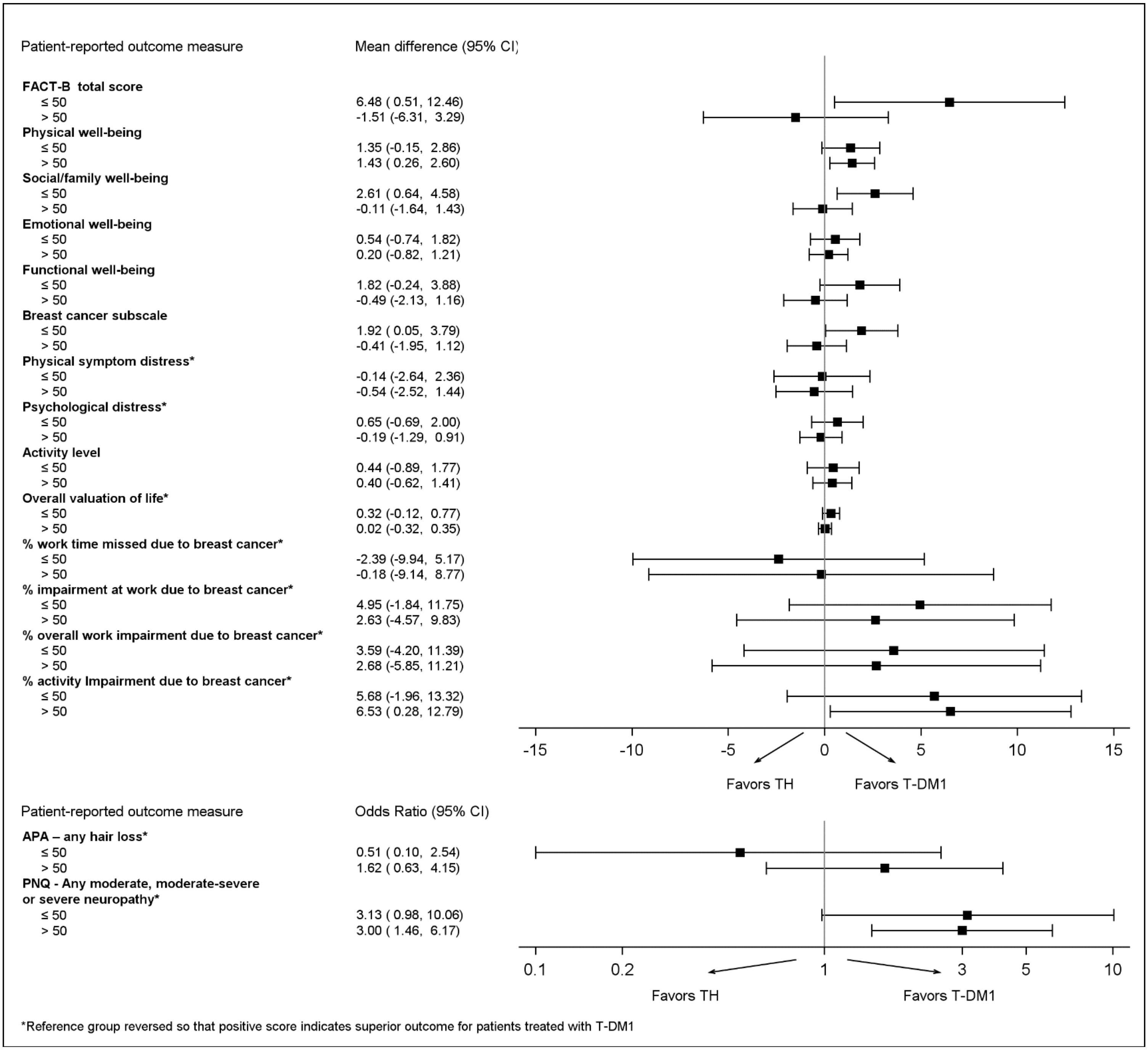
Patient-reported outcome measure
Mean difference (95% CI)
FACT-B total score
≤ 50 6.48 (0.51, 12.46)
> 50 -1.51 (-6.31, 3.29)
Physical well-being
≤ 50 1.35 (-0.15, 2.86)
> 50 1.43 (0.26, 2.60)
Social/family well-being
≤ 50 2.61 (0.64, 4.58)
> 50 -0.11 (-1.64, 1.43)
Emotional well-being
≤ 50 0.54 (-0.74, 1.82)
> 50 0.20 (-0.82, 1.21)
Functional well-being
≤ 50 1.82 (-0.24, 3.88)
> 50 -0.49 (-2.13, 1.16)
Breast cancer subscale
≤ 50 1.92 (0.05, 3.79)
> 50 -0.41 (-1.95, 1.12)
Physical symptom distress*
≤ 50 -0.14 (-2.64, 2.36)
> 50 -0.54 (-2.52, 1.44)
Psychological distress*
≤ 50 0.65 (-0.69, 2.00)
> 50 -0.19 (-1.29, 0.91)
Activity level
≤ 50 0.44 (-0.89, 1.77)
> 50 0.40 (-0.62, 1.41)
Overall valuation of life*
≤ 50 0.32 (-0.12, 0.77)
> 50 0.02 (-0.32, 0.35)
% work time missed due to breast cancer*
≤ 50 -2.39 (-9.94, 5.17)
> 50 -0.18 (-9.14, 8.77)
% impairment at work due to breast cancer*
≤ 50 4.95 (-1.84, 11.75)
> 50 2.63 (-4.57, 9.83)
% overall work impairment due to breast cancer*
≤ 50 3.59 (-4.20, 11.39)
> 50 2.68 (-5.85, 11.21)
% activity Impairment due to breast cancer*
≤ 50 5.68 (-1.96, 13.32)
> 50 6.53 (0.28, 12.79)
-15 -10 -5 0 5 10 15
Favors TH
Favors T-DM1
Patient-reported outcome measure
Odds Ratio (95% CI)
APA – any hair loss*
≤ 50 0.51 (0.10, 2.54)
> 50 1.62 (0.63, 4.15)
PNQ - Any moderate, moderate-severe or severe neuropathy*
≤ 50 3.13 (0.98, 10.06)
> 50 3.00 (1.46, 6.17)
0.1 0.2 1 3 5 10
Favors TH
Favors T-DM1
*Reference group reversed so that positive score indicates superior outcome for patients treated with T-DM1

Supplementary Note: List of Participating Institutional Review Boards (IRBs)

The following sites approved this study:

1. Dana Farber/ Harvard Cancer Center
2. Sarah Cannon Research Institute
3. University of North Carolina
4. Eastern Maine Medical Center (Northern Light)
5. Indiana University Washington University (St. Louis)
6. The Ohio State University
7. University of California, San Francisco
8. Johns Hopkins University
9. MD Anderson Cancer Center
10. St Luke Mountain State Tumor Institute
11. Loyola University
12. Memorial Sloan Kettering Cancer Center
13. Duke University Medical Center
14. North Shore-Long Island Jewish Health System
15. Baylor College of Medicine
16. Georgetown University
17. University of Chicago
18. The Mayo Clinic Cancer Center
19. University of Michigan
20. University of Pittsburgh Medical Center
21. University of Washington
22. University of Alabama
23. Georgetown University
24. Vanderbilt University
